# Comparative *in vivo* evaluation of cross-linked and non-cross-linked collagen membranes for guided bone regeneration in rat calvarial defects

**DOI:** 10.1590/1807-3107bor-2025.vol39.110

**Published:** 2025-11-07

**Authors:** Ana Maira Pereira BAGGIO, Arthur Henrique Alécio VIOTTO, Izabela Fornazari DELAMURA, Vinicius Ferreira BIZELLI, Ricardo Garcia Mureb JACOB, Ciro Borges Duailibe de DEUS, Rodrigo Faria NEIVA, Leonardo Perez FAVERANI, Ana Paula Farnezi BASSI

**Affiliations:** (a) Universidade Estadual Paulista – Unesp, Aracatuba Dental School, Department of Diagnosis and Surgery, Araçatuba, SP, Brazil.

**Keywords:** Bone Regeneration, Membranes, Biocompatible Materials

## Abstract

Guided bone regeneration (GBR) is a critical strategy for repairing large bone defects. This study aimed to assess the osteopromotive potential of a porcine cross-linked collagen membrane in critical calvaria-defects in rats. Seventy-two rats were divided into three groups: blood clot (CG - negative control), Bio-Gide^®^ membrane (BG - positive control), and Ossix Plus^®^ membrane (OSX - test). The defects were covered according to each group, and the experimental times were 7, 15, 30, and 60 days postoperatively. The collected samples were evaluated by histometric analysis, inflammatory profile, immunohistochemistry, and micro-computed tomography (micro-CT). At 7 days, no significant differences in bone neoformation were observed. At 15 days, the OSX group showed increased new bone formation compared to CG (p = 0.031). At 30 days, the BG group exhibited the most significant increase compared to both OSX (p < 0.001) and CG (p < 0.001). At 60 days, OSX demonstrated the highest osteopromotive potential, significantly outperforming CG (p < 0.001) and BG (p < 0.001). MicroCT analysis revealed that the OSX group had a bone volume (BV) of 17.33 ± 1.74 mm^3^, significantly higher than BG (8.06 ± 1.26 mm^3^, p < 0.05). The cross-linked collagen membrane was biologically more favorable for bone regeneration being a promising option for GBR procedures.

## Introduction

Guided bone regeneration (GBR) was established in 1980 and is currently considered a standard therapeutic procedure for the regeneration of bone defects in Implantology and Oral and Maxillofacial Surgery (OMFS).^1^ In GBR, the defect is covered by a membrane that acts as a barrier. It creates a favorable microenvironment for the repopulation of osteoprogenitor cells in the bone substitute to guide bone repair and prevent the growth of soft tissue.^2^

Ideal membranes for GBR should be biocompatible, resorbable, cell-occlusive, and osteopromotive, i.e., capable of supporting or enhancing bone tissue formation.^3^ Additionally, they should promote vascularization and allow nutrient diffusion.^4^

Currently, resorbable collagen membranes of porcine or bovine origin are the most commonly used in GBR procedures.^5^ These membranes may be chemically cross-linked to improve mechanical strength and enzymatic resistance. Membranes that are not cross-linked tend to degrade prematurely in vivo, compromising their barrier function.^4,6^ Cross-linking methods such as enzymatic glycation using sugars like ribose have been proposed to enhance the mechanical and biological stability of collagen-based membranes.^1^

Among these materials, some cross-linked collagen membranes have been designed to maintain their structural integrity for extended periods, allowing longer barrier function. These membranes are typically produced by isolating monomeric collagen, reconstituting collagen fibrils, and then chemically modifying them via glycation processes to increase resistance to enzymatic degradation.^1,7^ This process involves purification steps to remove immunogenic components, followed by reconstitution of collagen fibrils and cross-linking to improve resistance to enzymatic degradation. Preclinical data suggest that sugar-mediated cross-linking can extend membrane stability in vivo, potentially maintaining its barrier function for several months.^1,7^ These membranes are generally designed to be cell-occlusive while allowing the diffusion of fluids and plasma proteins, supporting tissue integration and nutrient exchange.^1,7^

Despite the promising physicochemical properties, there remains a lack of independent, peer-reviewed preclinical studies evaluating the osteopromotive potential of these membranes. Therefore, the present study aimed to assess the bone regeneration capacity of a porcine-derived cross-linked collagen membrane in critical-size calvarial defects in rats.

## Methods

### Experimental design

The research was approved by the Animal Research Ethics Committee of the Faculty of Dentistry of Aracatuba – FOA UNESP, under protocol number 0402-2022 and was conducted following the principles of the ARRIVE Guidelines.

**Figure 1:**
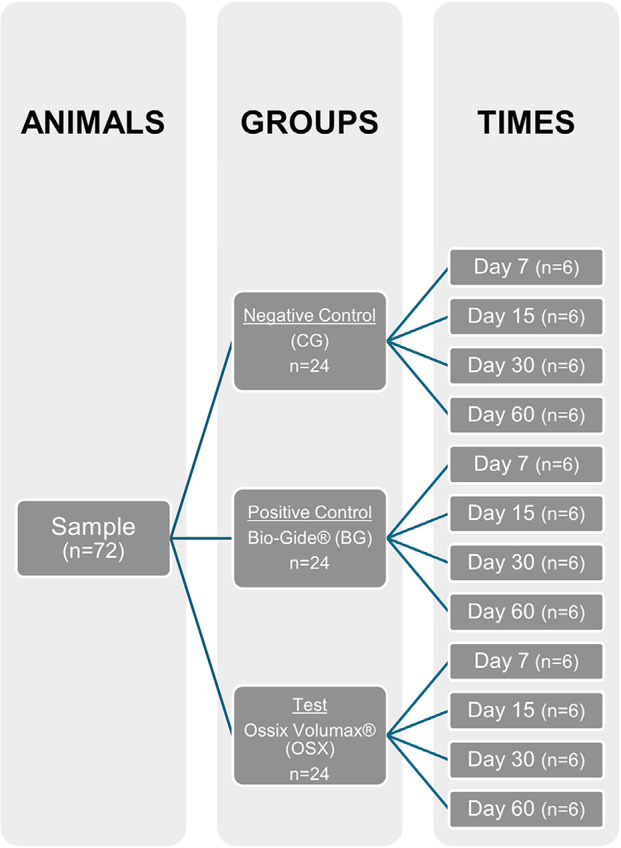
Experimental groups and time points.

A total of 72 male rats (Rattus norvegicus albinus Wistar), aged 3 to 4 months and weighing approximately 300 g were used in the study. The animals were randomly divided into three groups (n = 6 per group) for each time point analyzed (7, 15, 30, and 60 days). The number of animals per group was calculated based on a previous study that estimated a minimum difference of 27.5% in bone neoformation, with an expected standard deviation of 13.8%, a test power of 80%, and p > 0.005, resulting in n=6 animals per group^8,9^ (Figure 1).

Each group received the following treatment: CG Group (negative control), BG Group (positive control with Bio-Gide^®^), and OSX Group (cross-linked collagen membrane).

The animals were housed in collective cages at the Animal Facility of the São Paulo State University – UNESP, with three animals per cage, under controlled conditions of temperature (22 ± 2°C) and relative humidity (60 ± 10%) and a 12-hour light/dark cycle. They were provided with a balanced diet and *ad libitum* access to water throughout the experimental period.

### Bio-Gide**®** collagen membranes (positive control)

Two Bio-Gide^®^ collagen membranes measuring 30×40 mm were cut into 10×10 mm sections using a millimeter ruler to match the critical sized bone defect of 8 mm in diameter, totaling 24 membranes for the experiment (Bio-Gide^®^, Geistlich Biomaterials, Switzerland).

### Ossix Plus**®** collagen membranes (test)

Six Ossix Plus^®^ collagen membranes measuring 10×40 mm were cut into 10×10 mm sections using a millimeter ruler to match the critical defect of 8 mm in diameter, totaling 24 membranes for the experiment (Ossix Plus^®^, Datum Dental Ltd, Telrad, Israel).

### Surgical procedure

The surgical procedure followed the standard method of previous studies by this research group^9,10^with modifications to suit the study objectives.

**Figure 2:**
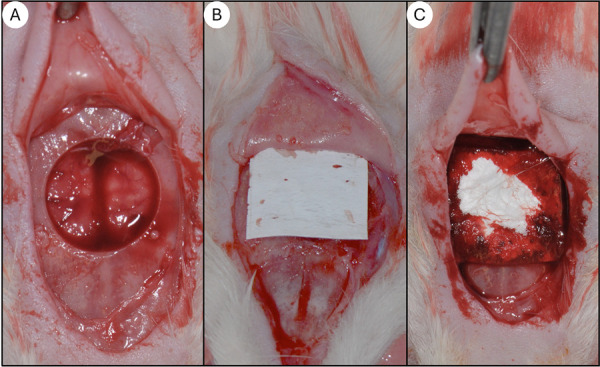
Representative images of critical-sized calvaria bone defects in rats. (a) Bone defect without membrane coverage (blood clot group). (b) Bone defect covered with a cross-linked collagen membrane (Ossix Plus). (c) Bone defect covered with a non-cross-linked collagen membrane (Bio-Gide).

Briefly, after anesthetizing the animals with an intraperitoneal injection of a combination of xylazine (10 mg/kg) and ketamine (80 mg/kg), the animals were positioned on a heated surgical table to maintain body temperature. A midline incision was made on the scalp to expose the calvaria, and a critical-sized bone defect of 8 mm in diameter was created using a low-speed trephine drill, as previously described^9,10^(Figure 2).

The surgical site was thoroughly irrigated with saline solution to minimize heat-induced tissue damage. After the defect was created, the membrane corresponding to the respective group (BG or OSX) was carefully placed over the calvarial defect, ensuring full coverage of the area. The animals of the CG group did not receive any membrane, but the bone defect was created using the same protocol. The periosteum was repositioned, and the skin was sutured in layers using 4-0 silk sutures. In the immediate postoperative period, each animal received a single intramuscular dose of 0.2 mL of penicillin G benzathine. The animals were monitored daily for any signs of infection or complications.

At the designated time points (7, 15, 30, and 60 days), the animals were euthanized by an overdose of xylazine and ketamine, and the calvarias were carefully harvested. The calvarial samples were fixed in 10% formaldehyde for 48 hours and then decalcified in 10% ethylenediaminetetraacetic acid (EDTA) for 5 weeks before further processing for histological and imaging analyses.

Any animals that experienced significant intraoperative or postoperative complications, such as infection or membrane displacement, were excluded from the study.

## Analyses

### Histological and histometric analysis

The slides were prepared according to Bassi et al.^7^ The histological evaluation was conducted to verify bone neoformation, presence of inflammatory infiltrate, formation of connective tissue, and membrane degradation.

The samples were coded by a single blinded examiner who was unaware of the groups that each animal belonged to. Measurements were performed using an optical microscope (LeicaR DMLB, Heerbrugg, Switzerland) equipped with an image capture camera (LeicaR DC 300F Microsystems Ltd, Heerbrugg, Switzerland) connected to a computer with the digital image analysis software ImageJ (Image Processing and Analysis Software, National Institutes of Health, Bethesda, Maryland). The digitized images were saved in JPEG format for later analysis. The area of bone tissue in the central region of the defects was evaluated, and the data obtained were converted from absolute pixel values to relative percentage values, evaluated according to Bizelli et al.^10^

### Quantification of inflammatory cells and blood vessels

To assess the local inflammatory response and vascularization, histological quantification of inflammatory cells and blood vessels was performed. For each animal and at each experimental time point, one histological slide was randomly selected. Two tissue sections per slide were photographed using a light microscope (DM 4000B, Leica) equipped with a color image processor (Leica Qwin V3 software) and a digital camera (DFC 500, Leica). Images were acquired from three standardized regions within the defect: central region, right margin, and left margin.

Using ImageJ software at 100× magnification, a grid with 130 intersecting points was applied to each image. Cells and blood vessels intersecting the grid points were manually counted. The results were expressed as the number of inflammatory cells or vessels per grid area, enabling a semi-quantitative comparison between groups.

### Immunohistochemistry

Slides containing tissue sections were prepared and treated with hydrogen peroxide to neutralize endogenous peroxidase activity. Half of the slides underwent antigen retrieval to improve antigen exposure, followed by blocking of endogenous biotin. Specific primary antibodies against osteocalcin (OC) and osteopontin (OP) were used, followed by biotinylated secondary antibodies. To increase detection sensitivity, we employed an amplification system using Avidin and Biotin. Protein visualization was performed using the chromogen diaminobenzidine (DAB), which produces a brown coloration where immunostaining is present.

Subsequently, the sections were counterstained with Harris hematoxylin to enhance contrast and facilitate visualization under the optical microscope. Protein expression was evaluated semi quantitatively, assigning scores from 0 to 3 based on the intensity and extent of immunostaining. This method allows a detailed analysis of the presence and distribution of OC and OP during different stages of the bone repair process, providing valuable insights into the dynamics of these proteins in this biological context.

### Micro-computed tomography (Micro-CT)

The parameters used were as follows: pixel size 11.87 μm, 50 kVp, 0.5 mm aluminum filter, 0.6° rotation, and 180° arc rotation. After scanning, the obtained images were imported into the NRecon Reconstruction software (Skyscan, Bruker, Kontich, Belgium) for three-dimensional (3D) reconstruction of the calvaria in grayscale. After obtaining the 3D images, the Data-Viewer software (Skyscan, Bruker, Kontich, Belgium) was used to determine the volume of interest, which was standardized for all images in the coronal section. The obtained sections were imported into the CT-Analyzer software (version 1.14.4, Skyscan, Bruker, Kontich, Belgium) to evaluate morphometric parameters, such as bone volume (BV), bone volume fraction (BV/TV), trabecular thickness (Tb.Th), trabecular number (Tb.N), trabecular separation (Tb.Sp), and total bone porosity percentage (Po.tot) (Skyscan, Bruker, Kontich, Belgium). The region of interest was delineated according to the rounded morphology of the defects, which was also standardized for all reconstructions (9.74 × 9.74). Subsequently, grayscale values ranging from 105 to 242 in 40 layers were used. The images were then converted to grayscale for calculating 3D parameters in millimeters (mm) using the CT Analyzer software.

### Statistical Analysis

Statistical analysis was performed using SigmaPlot version 12.0 (Systat Software Inc., San Jose, USA). Data were tested for normality using the Shapiro-Wilk test. For comparisons between groups at each time point, one-way ANOVA was used followed by Tukey’s post hoc test for multiple comparisons. When normality was not confirmed, the Kruskal-Wallis test followed by Dunn’s post hoc test was applied.

Quantitative data were expressed as mean ± standard deviation (SD). A significance level of p < 0.05 was adopted for all statistical tests. Each time point was analyzed independently, and there were no repeated measures in the study design. All statistical analyses were performed with a confidence interval of 95%.

## Results

### Histological and histometric analysis

At 7 days of bone repair, all groups behaved similarly regarding bone neoformation, with no statistical differences among any of them. At 15 days, despite a greater amount of newly formed bone observed in the CG group, only the OSX group showed a statistical difference compared to the CG group (p = 0.031).

During a later stage of the bone repair process (30 days), the CG group continued to perform as expected, and the BG group had the most significant increase in the amount of newly formed bone, showing statistical differences compared to the OSX (p < 0.001) and CG (p < 0.001) groups. The OSX group, despite showing an increase in bone neoformation, did not show a statistical difference compared to the negative control group CG.

**Figure 3:**
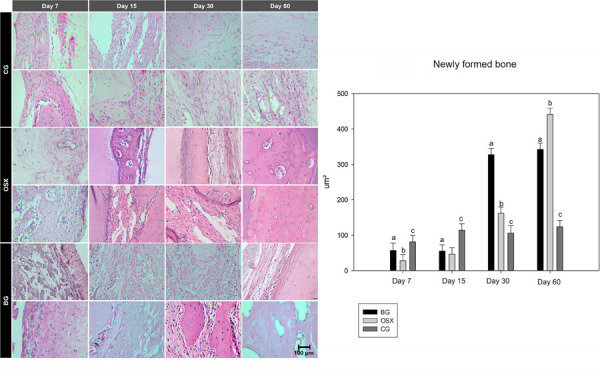
Representative photomicrographs (x40) ) and graph comparing the areas of bone neoformation between the groups (BG, OSX, and CG) and periods analyzed (7, 15, 30, and 60 days).

In the final experimental time point at 60 days, the OSX group demonstrated the best osteopromotive potential, with significant differences compared to the CG (p < 0.001) and BG (p < 0.001) groups, with the BG group also showing a statistical difference compared to the CG group (p < 0.001) (Figure 3).

### Quantification of inflammatory cells and blood vessels

**Figure 4:**
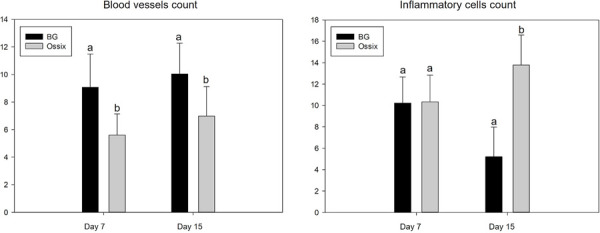
Average number of inflammatory cells and blood vessels (BG and OSX groups) at 7 and 15 days.

In an intragroup comparative analysis of the lymphocyte count, no statistical differences were found when analyzing the time factor from 7 to 15 days (p = 0.539). However, for the membrane factor, a statistical difference was found (p = 0.004). In the intergroup comparative analysis at the 7-day time-point, the BG and OSX groups showed similar results in the quantity of cellular content, with no statistical difference (p = 0.953). When analyzing the evolution of the repair process at 15 days, the BG group showed a milder inflammatory response, while the OSX group showed a significant increase in cellular content (p < 0.001) (Figure 4).

Evaluating the angiogenic capacity of the membranes, an intragroup comparative analysis showed that only the membrane factor significantly influenced the inflammatory process (p = 0.043) during the evolution of the repair process from 7 to 15 days. In the intergroup analysis at 7 and 15 days, despite similar behavior between the BG and OSX groups, a greater number of blood vessels was found in the BG group. Both groups showed an increase in blood vessels during the repair evolution, with no statistical difference (p = 0.122 and p = 0.153) (Figure 4).

**Figure 5:**
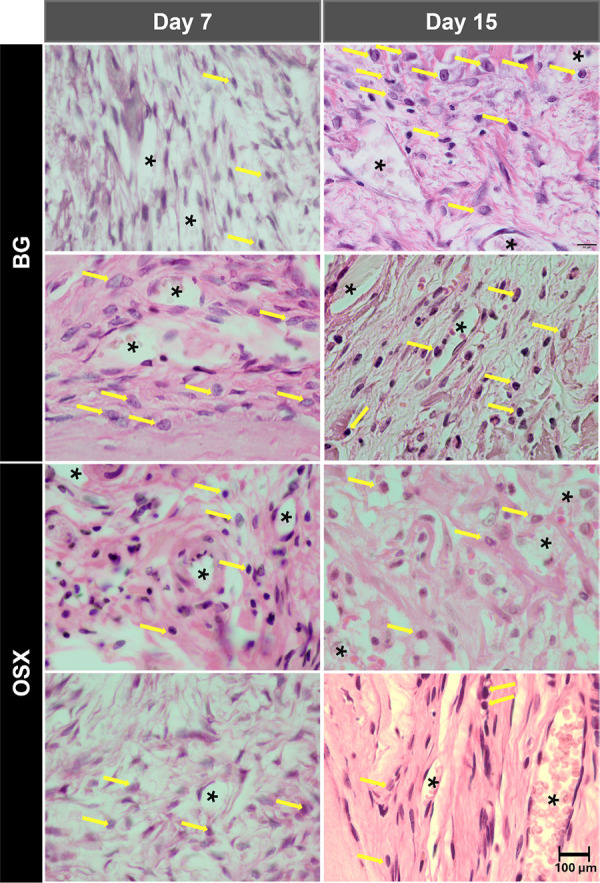
Photomicrographs (×100) demonstrating the average number of inflammatory cells and blood vessels (BG and OSX groups) at 7 and 15 days. *Blood vessels; yellow arrows: inflammatory cells.

Figure 5 shows photomicrographs (×100) demonstrating inflammatory cells and blood vessels (BG and OSX groups) at 7 and 15 days.

### Immunohistochemical analysis

#### Osteocalcin

**Figure 6:**
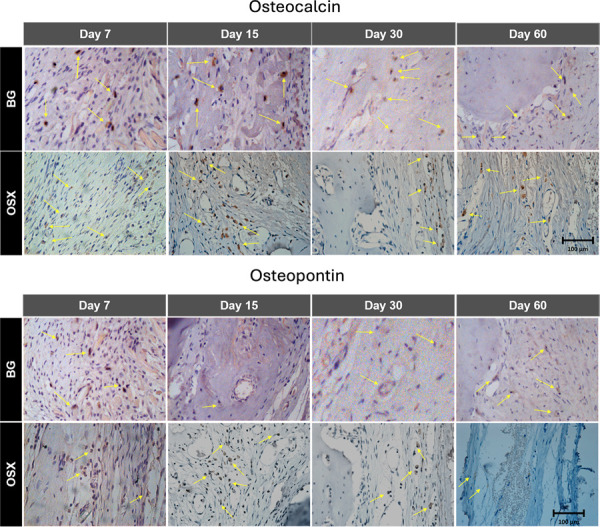
Photomicrographs of osteocalcin and osteopontin immunostaining at 7, 15, 30, and 60 days. Yellow arrows indicate expression. Magnification: 40x.

In a semiquantitative comparison, the BG group showed positive immunostaining for OC only at the initial time point (7 days) and hyperpositive staining in the other experimental time points (15, 30, and 60 days). The OSX group, however, showed intense staining in all periods (Figure 6).

#### Osteopontin

Both the BG and OSX groups exhibited hyperpositive immunostaining for OP at 7 and 15 days, and positive immunostaining at 30 and 60 days (Figure 6).

#### MicroCT analysis

**Figure 7:**
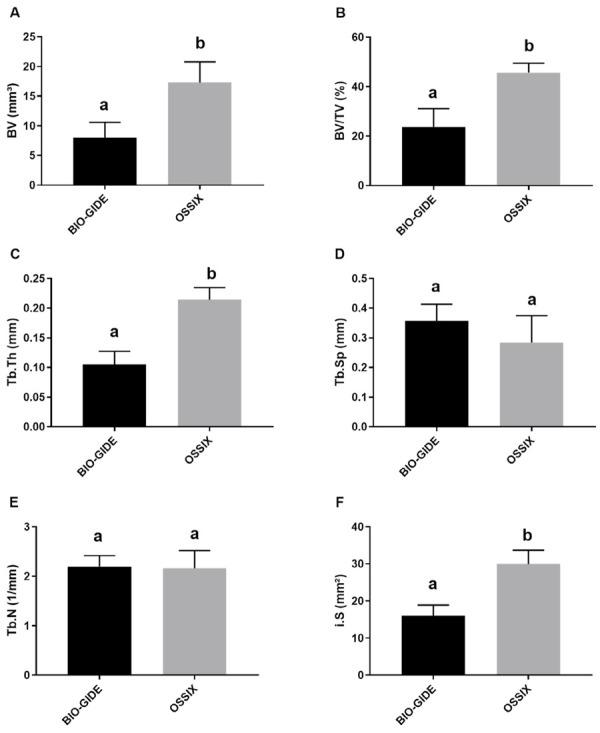
Graphs of morphometric results. Mean values and standard deviation for BV (A), BV/TV (B), Tb.Th (C), Tb.Sp (D), Tb.N (E), and i.S (F).

**Figure 8:**
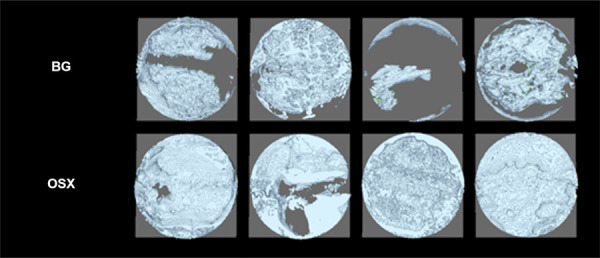
3D microtomographic reconstructions of the critical defect region at 60 days. Line 01: BG and Line 02: OSX.

All MicroCT parameters are represented in Figure 5. Regarding the measurement of BV, the OSX group exhibited a value of 17.33 ± 1.74 mm^3^, whereas the BG group had a value of 8.06 ± 1.26 mm^3^ (p < 0.05). Evaluating BV/TV, the OSX group showed a higher value with a mean of 45.67%, compared to a lower value for the BG group at 23.69% (p < 0.05). In quantifying Tb.Th results, the experimental group (OSX) demonstrated promising numbers with 0.214 mm, whereas the BG control group showed 0.105 mm (p < 0.05). Measurements of Tb.Sp did not show significant differences between the compared groups, with averages of 0.285 mm for OSX and 0.357 mm for BG (p > 0.05). For Tb.N, values were similar between the two membranes, with OSX at 2.162 1/mm and a slight increase noted for BG at 2.199 1/mm (p > 0.05). Regarding the quantification of i.S (intersection surface), there was a significant difference in the results (p < 0.05), with values of 29.97 mm^2^ for OSX and 16.05 mm^2^ for BG (Figure 7). Figure 8 shows 3D microtomographic reconstructions of the critical defect region at 60 days.

## Discussion

The main goal of this study was to analyze the osteopromotive behavior of a collagen membrane derived from porcine tendon, treated with a new collagen rectification method involving sugar glycation. This treatment aims to enhance the membrane’s mechanical strength and maintain its function as a physical barrier for a longer period, while minimizing intense inflammatory processes. The treatment also facilitates cellular and angiogenic permeability to promote new bone formation. The commercially established Bio-Gide^®^ membrane, widely used in regenerative process, served as the control for comparison.

The results of this study revealed important differences in the biological behavior of the analyzed membranes, corroborating some observations of previous studies. The Bio-Gide^®^ membrane has proven effective in several studies due to its favorable biological properties, including biocompatibility and its ability to promote angiogenesis.^11-13^ This favorable behavior aligns with observations that collagenase, the main component of Bio-Gide^®^ membranes, is highly biocompatible and promotes bone regeneration by inducing an anti-inflammatory environment conducive to healing.^14-16^ However, our study also observed that, although Bio-Gide^®^ showed excellent biological performance in the early stages of regeneration, the Ossix^®^ membrane with sugar glycation demonstrated superior performance in bone formation, especially at later stages (30–60 days)

The porcine collagen membrane Bio-Gide^®^ does not undergo collagen rectification, which is a chemical treatment to extend membrane activity, thus enhancing its biocompatibility. This aspect is clearly reflected in the results of the inflammatory profile analysis, showing a low index of inflammatory cells at 7 days, which decreases by 15 days. Another desirable characteristic of a good membrane is the promotion of blood vessels proliferation, precisely the behavior observed in this study and supported by another research.^17-19^ Due to its favorable initial biological behavior, Bio-Gide^®^ facilitates physiological bone neoformation, resulting in near-complete closure of critical-sized with newly formed bone tissue. These effects were evidenced both through immunostaining, demonstrating increased osteopromotion at 7 and 15 days, and the prevalence of osteocalcin at 30 and 60 days. These more orderly processes contribute to the rapid and substantial bone formation observed withBio-Gide^®^.

The Ossix^®^ membrane, in comparison, presented a slightly higher inflammatory profile in the early days but with a favorable response characterized by a reduction in inflammation and an increase in angiogenesis between days 7 and 15. This suggests that collagen glycation may have a long-term modulatory effect on inflammatory and angiogenic responses, allowing for a more favorable environment for bone regeneration in the long run. The study by Radenkovic et al.^23^ also highlights the importance of glycation modules as a factor in increased bone regeneration, although the long-term effects of this modulation still require further investigation.

Moreover, when analyzing the biological mechanisms involved, we observed that the greater bone formation seen in the Ossix^®^ membranes may be related to the influence of glycation in cellular signaling pathways, particularly vascular endothelial growth factor (VEGF) and transcription factors related to osteogenesis. Glycation can enhance the integrity and stability of the extracellular matrix, promoting a more robust organization of bone tissue in the early stages of healing.^21,22^

Ossix^®^ is a non-porous material, providing an exclusive barrier function. In contrast, Bio-Gide^®^ consists of two distinct layers: one compact and the other loose and porous. Understanding the original condition of each barrier membrane provides insight into their integration patterns, which include cellular infiltration, vascularization, and degradation—essential properties for GBR. These variations can influence clinical outcomes of bone regeneration and augmentation. Histological analysis of the two membranes revealed that the integration pattern of Ossix^®^ is completely different from that of Bio-Gide^®^ since it does not integrate with the surrounding tissue. The primary functionality of a GBR membrane is space maintenance, which was achieved effectively by both membranes analyzed, as they remained stable without any signs of fragmentation up to 30 days. However, recent requirements for collagen-based GBR membranes include transmembrane vascularization. Some studies indicate that sugar crosslinked membranes may not support neo-angiogenesis, potentially reducing their regenerative potential compared to conventional collagen-based membranes.^21^

However, research on the macrophage rate of its two phenotypes, M1 (pro-inflammatory) and M2 (anti-inflammatory), reveal excellent biocompatibility and regenerative potential of the Ossix^®^ membrane. This is because macrophages play a crucial role in bone regeneration, and according to Toledano et al.,^12^ achieving a shift from the M1 to M2 phenotype would result in bone regeneration due to an anti-inflammatory environment favorable to the regenerative process. This was observed in the study by Radenkovic,^23^ where the M2 cell count in the Ossix^®^ membrane implant region was higher than in Bio-Gide^®^ over the course of the experiment, promoting greater bone neoformation. The favoring of M2 differentiation and quantification when using the Ossix^®^ membrane may explain the higher inflammatory profile in the Ossix^®^ group. This assumption is mainly supported by the greater bone formation achieved by the Ossix^®^ test group at the end of the experimental periods compared to the control group using Bio-Gide^®^.

Another point to highlight is the long-term stability without any cellular or tissue growth on the Ossix^®^ membrane, unlike the integration behavior observed with the Bio-Gide^®^ membrane. These results should be further examined in new in vivo studies, as this tissue integration contradicts previous assumptions, including the theory that more bioactive membranes that support processes such as transmembrane vascularization should be used to support the regeneration process.^23,24^ In this study, we observed membrane integrity of Ossix^®^ at 60 days with still high values of OC, which may indicate that the bone neoformation process was still under way.

The favorable behavior of the Ossix^®^ membrane regarding bone neoformation in this research occurred even with less neoangiogenesis compared to Bio-Gide^®^, as evaluated through histometric and micro-CT results at 60 days. Micro-CT analysis allows quantification of regenerative gains. The Bio-Gide^®^ membrane, consistent with other studies, demonstrated excellent performance in the bone repair of the critical defect, but the Ossix^®^ membrane provided a greater bone volume. Higher means were detected for the Ossix^®^ group in parameters such as BV, BV/TV, Tb.Th, and i.S (p < 0.05), resulting in greater quantity and quality of bone, but no significant differences were found for Tb.Sp and Tb.N analyses (p > 0.05) compared to Bio-Gide^®^. This is consistent with the observations of Zubery et al.,^25^ who suggest that glycation membranes promote greater stability and mechanical rigidity, contributing to better bone integration.

Bone defects are considered critical when they do not regenerate spontaneously throughout the animal’s life. Creating a non-self-repairing bone lesion is a valuable evaluative means for assessing the biological performance of biomaterials.^24,26,27^ The repair of a bone defect filled with a clot without the application of a membrane occurs in a standardized manner, with newly formed bone around its periphery and fibrous connective tissue in the center, as observed in the negative control group. By placing a membrane, it is possible to protect the central region of the defect, resulting in a notable increase in newly formed bone as the organization and structure of the clot is maintained in place, which favors satisfactory repair.^27,28^ This observation aligns with the present study, as both membranes provided significant bone neoformation, with almost complete defect obliteration observed for Ossix^®^.

The examined membranes are originated from the same animal species but are obtained from different tissues and undergo different manufacturing processes, leading to the differences in the integration behavior of the cross-linked sugar collagen membrane compared to the native collagen membrane. This suggests that the cross-linked sugar collagen membrane should only be used as a barrier membrane.

Despite small differences, both membranes create a suitable environment for GBR. Further preclinical in vivo studies should be conducted to help demonstrate the exact differences between these membranes in bone regeneration. However, the current results lead to the overall conclusion that both membranes are suitable for bone tissue regeneration. The choice of a membrane should be made based on clinical needs and must present basic properties such as biocompatibility, mechanical strength, tissue integration, partial cell occlusion, ease of handling and installation, availability, and patient accessibility. For successful GBR, the membrane needs to remain intact for a sufficient period of time to allow for osteoid tissue formation^17,24,31^.

### Study Limitations

While the results of this study provide valuable insights into the osteopromotive behavior of collagen membranes, there are several limitations that should be considered. First, the animal model used in this study is a commonly accepted model for bone regeneration, but it does not fully replicate the complexity of human bone healing processes. Differences in healing dynamics between species could influence the direct translation of these findings to clinical settings.

Secondly, the study did not include a graft material or scaffold in the bone defects, which could have influenced the regeneration process. Grafting materials are commonly used in clinical practice to support bone healing and might have contributed to the overall regeneration observed. The absence of such materials may limit the applicability of our findings to clinical scenarios where scaffolds or grafts are involved in bone repair.

## Conclusions

In this preclinical study using a non-grafted animal model, a cross-linked collagen membrane obtained through glycation demonstrated better bone regeneration compared than a non-cross-linked collagen membrane, particularly at later time points (30–60 days). Histological and micro-CT analyses revealed greater bone volume and improved structural organization associated with the cross-linked membrane, along with increased osteocalcin expression. Although an initial inflammatory response was observed, it diminished over time, and angiogenic activity was maintained. The non-cross-linked membrane showed favorable early outcomes but did not achieve the same level of long-term bone formation. These findings suggest that cross-linking via glycation may contribute to improved membrane stability and bone regenerative potential. However, further studies are required to confirm these results in clinical settings, particularly under standard bone regeneration protocols involving graft materials.

## Data Availability

The authors declare that all data generated or analyzed during this study are included in this published article.
